# The moral economy of home construction in late socialist Yugoslavia

**DOI:** 10.1080/02757206.2017.1340279

**Published:** 2017-06-26

**Authors:** Rory Archer

**Keywords:** Socialist working class, Yugoslavia, moral economy, informal construction, housing

## Abstract

Housing shortages in Yugoslav cities were a perennial concern for authorities and citizens alike. They disproportionately affected Yugoslav workers who as a consequence were the demographic most likely to independently construct a family home. This article explores how informal builders justified home construction in moral terms, legitimizing it on the basis of physical labour that was invested in home construction. This was couched in both the language register of Yugoslav socialism *and* patriarchal custom (according to which a male-headed household should enjoy the right to a family home). Construction was also conditioned by the opportunities and constraints of late socialist temporalities.

Heavily subsidized housing, usually in the form of high-rise apartment buildings, was foreseen by urban planners to be the most desirable and egalitarian way to house the ever-increasing number of rural-to-urban migrants in Yugoslav cities after World War II (Le Normand 2012, 353–357) as in other socialist states (Andrusz 1984; Gentile and Sjöberg 2013). In practice, however, high building costs and the huge demand for flats rendered the independently built family home a cornerstone of Yugoslav housing provision. Although transformative urban projects like the construction of Novi Beograd were undertaken (Le Normand 2014), Yugoslavia never attempted to house its inhabitants in the ambitious, utopian scale that the Soviet Union achieved between 1956 and 1965 when a third of the population was housed in newly constructed apartments (Reid 2014, 89) and the authorities proclaimed that housing shortages would be solved within 10 or 12 years (Harris 2013, 9). In terms of housing provision, Yugoslavia had more in common with neighbouring Albania (Dalakoglou 2017, 133–135), Hungary (Molnár 2010, 62; Fehérváry 2013) and Bulgaria (Tsenkova 2009, 42–43) where despite a cultural and civilizational distaste for family homes according to socialist sensibilities, home building nevertheless surpassed state sector apartments. Although apartments were ‘privileged as the proper form of socialist living’ (Fehérváry 2013, 76), individuals were encouraged to construct homes independently in order to shift the burden of home provision away from the state. With the shift to worker self-management in the early 1950s in Yugoslavia (Unkovski-Korica 2016), public housing became part of the socially owned sector.

Yugoslavia’s blue-collar workers were less likely to receive a socially owned flat than white-collar and skilled workers who were systemically favoured in the allocation process (Živković 1968; Bobić and Vujović 1985; Petrović 2004; Le Normand 2012; Archer 2016). Housing allocation thus forged novel social inequalities, a phenomenon observed by scholars in other state-socialist contexts (Szelenyi 1983; Bodnár and Böröcz 1998; Kulu 2003; Pelikánová 2012; Fehérváry 2013, 74–75; Gentile and Sjöberg 2013; Harris 2013, 112; Dalakoglou 2017, 137). In contrast to capitalist societies where wealthier strata were more likely to own their own home, in socialist states like Yugoslavia white-collar employees and managers were significantly *less* likely to own property than poorer workers, instead enjoying the more prestigious use of a socially owned flat. This was arguably more advantageous than property ownership as socially owned flats ‘ … were acquired without incurring personal costs, while in terms of their use, inheritance and even trading there were practically no differences between the holders of tenancy rights to state/socially owned housing and private owners’ (Petrović 2004, 270).

In the absence of conditions to house the majority of workers in socially owned flats, workplaces provided credit to facilitate the individual construction of houses in urban peripheries. Predominantly with their own embodied labour (and that of families, friends and neighbours) individuals constructed family homes across Yugoslavia’s cities and towns. This article explores how workers – the largest group of independent homebuilders – justified construction by making claims of deservingness with a particular emphasis on the physical labour invested in home construction. Methodologically, I draw upon oral history research undertaken with 30 Belgraders about their experience of housing and the workplace in late socialism. This is complemented by archival documents from the narrators’ workplaces, factory newspapers and print media.^1^

Although limited ownership of private property was permitted in Yugoslav socialism independent builders nevertheless encountered a number of potential moral and legal quagmires. These included the stigma of private property and rent seeking according to socialist ideology, the practical difficulties of arranging for construction entirely within the letter of the law and cultural racism which was increasingly levelled against new migrants to the city who due to their rural origins were accused of failing to sufficiently adapt to the values of city life (for example, see Simić 1973, 126–147; Čolović 1984, 147; Vidić Rasmussen 1995, 241).

Yugoslav urban sociologists fretted during the 1970s and 1980s about the alienation of workers who built homes informally (for example, see Živković 1968, 1981; Bobić and Vujović 1985; Vujović 1986, 1987; on comparable debates in Hungary, see Fehérváry 2013, 80–81; Molnár 2010, 62–63). Some workers, however, described their satisfaction with the arrangement and preferred ownership of a self-built family home to the use of a socially owned flat.^2^ The construction process of independent family homes was simultaneously couched in the language register of Yugoslav socialism *and* patriarchal custom (according to which a male-headed household should enjoy the right to a family home). Workers at times even referred explicitly to an innate, patriarchal desire for a family house. Others, however, shared the view of sociologists in considering it to be patently unfair that Yugoslav workers were obliged to contribute to housing funds for their entire working lives while simultaneously financing an independently built family home.^3^

In addition to framing construction in moral terms, home building was conditioned by the particular temporalities of late socialism (Verdery 1996; Hanson 1997; Mihelj and Huxtable 2016). Home builders spoke about the ‘spirit of the time’ which they felt was encouraging to home builders. Indeed, the temporal frame of a ‘normal life’ in late socialist Yugoslavia parallels a Fordist framework of biographical progression accompanying temporal regimentations of production and consumption. Industrialization and market socialism by the late 1960s had for many Yugoslavs, ‘structured a life-cycle temporality that granted a narrative coherence to events and delineated an arc along which economic security could be pursued, professional progress could be measured, and markers of status and prestige could be granted value’ (Muehlebach and Shoshan 2012, 334). According to Koleva (2008, 28–30), a normal life course, embedded in the cultural code of socialism and endowed with normative power, not only proscribes the parameters of a ‘normal’ life course but ‘outlines the horizon of expectations related to what a good life should be like.’ Labour is represented at the core of such a biography, the ‘moral duty of the socialist citizen’ (Koleva 2008, see also Alheit and Dausien 1985; Dausien 1996).

The linear progression inherent to socialism remains a durable force in the post-socialist context. Jansen (2015, 44), in his study of contemporary temporal reasoning in Sarajevo, posits that yearnings for ‘normal lives’ in the context of Bosnian ‘spatiotemporal entrapment’ are overwhelmingly underscored by a linear, forward movement, embedded in modernism. Oral history narrators referred to the frustration they felt by the interruption of this linear, forward movement. For example, Branimir, a retired textile worker, reveals this sense of despondency: ‘I could never imagine that times like this would come. I was born in 1948 … and so you automatically think it will be eternally better. I did not consider that a time will come when it will not be.’^4^

As well as understanding housing in relation to a normative, linear life course, construction was also undertaken according to the particular temporal rhythm of the Yugoslav social sector workplace and the free time this provided (in the evenings, weekends and holidays). Financial arrangements (chiefly low interest loans) were often predicated on the assumption that high inflation would reduce repayments over time, particularly during the economic crisis after 1979. Narrators identified a moral economy between the state and its citizens in housing provision within which low interest loans in conditions of high inflation were of key importance. As Leutloff-Grandits (2006, 87) observes in rural socialist Croatia, either receiving a socially owned flat or building a home was considered to be a social entitlement. With the stock of flats severely limited, workers considered they had the right to construct a home independently. As in other state-socialist contexts, building took place in ‘an enduring, paternalistic paradigm for citizen-state relations’ (Fehérváry 2013, 76) within which Yugoslav authorities were ambivalent regarding home construction. The decentralized logic of Yugoslav socialism, however, meant that unlike other socialist states the most important actors involved in financing, regulating and sanctioning construction were workplaces^5^ and local municipalities. Thus, opportunities and constraints in home construction varied significantly between various Yugoslav workplaces, municipalities, cities and republics.

## ‘Rogue construction’

The pattern of independent home construction in Yugoslavia had changed with the swing to market socialism in the mid-1960s. While the earlier form it took was of individually built, dispersed and tattered homes, by the late 1960s and 1970s construction became denser as entire neighbourhoods were built, usually illegally but with the acquiescence of the authorities. During the 1970s and 1980s, larger Yugoslav cities experienced a ‘radical spatial and urban transformation’ with the rogue building of new settlements on the city fringes and in surrounding villages (Tanić 1989, 147). Such homes have been typologized as ‘redbrick architecture’, close to the ‘classical understanding of informalism, that is, dusk-till-dawn housing construction’, durable, with each floor allotted for a generation of the family (Topalović 2012, 101).

The semi-legal or illegal construction of single family homes in Yugoslav urban peripheries was known as wild building [*divlja gradnja*], unlicensed or illegal construction [*bespravna gradnja*]. Le Normand (2014, 148) considers these terms problematic in that ‘wild’ holds connotations of savagery and incivility while ‘illegal’ refers to an extremely broad spectrum of illegality. Thus she advocates for use of the term ‘rogue construction’ whereby the emphasis is on the fact that construction evaded the control of the authorities (Le Normand 2014). Despite developing beyond the reach of urban planners, rogue construction did receive varying degrees of tacit or direct support from the authorities and workplaces as a means to provide housing to workers without having to engage in large-scale public works. In official and media discourses of rogue construction, however, issues of the exploitation of workers would come to the fore.

The terms used were as much a way of framing the discussion on rogue construction, and the appropriate remedy to it, as a reflection on the true nature of the builders. Builders were characterized as social cases needing to be cared for and removed from public sight; speculators who should be disciplined and punished; nouveaux-riche who should be humbled. These were all cases of delinquency. Only in the case of exploited workers was it necessary to question the way in which the state functioned. (Le Normand 2014, 160–161)

Živković (1981, 235) considered it possible to explore rogue construction from a number of different perspectives – legally (as an expression of undisciplined citizens), in terms of urbanism (as an obstacle to the realization of urban planning) and sociologically. Sociologically, he considered that rogue construction was the ‘self-initiative of second-class citizens in resolving their housing situation’ because they did not receive a flat from society (and had no chance to access one) despite the collective financing of the flats which society gave to its ‘first-class citizens’. He thus considered illegal building in Marxist terms as empirical proof of the existence of the exploited and the exploiter, and neighbourhoods of illegal buildings as a visible form of social segregation (Živković 1981) (on urban segregation in Belgrade, see Archer 2016, 60–63).

The construction of such homes was most frequently neither entirely legal nor outright illegal but rather existed on a continuum. Some workers undertook homebuilding with utter disregard for planning regulations while others followed rules to the letter. The majority of builders, however, undertook home construction projects in the ambiguous space in between – not entirely legally (at least in part due to the confusing and often contradictory regulations) but also not wholly illegally. In the absence of an established home construction industry, independent home-building projects took place through the invocation of rural reciprocal obligations and the informal exchange of goods and services (similar dynamics have been detailed in Hungary [Sík 1988a, 1988b; Fehérváry 2013, 81] and Bulgaria [Creed 1998, 200–202]).

The work of municipal administration was identified as a particular hindrance for would-be legal builders in Yugoslavia (Jambrović 1980). Contradictory and vague laws and unobliging municipal officials contrived to make sticking to the letter of the law difficult for citizens (many of whom were unaware that they were infringing on it) (Le Normand 2014, 148). The process of legally building was so complicated that it was very likely that individuals, even with the best of intentions, would break the law in some fashion during the long and bureaucratic process of applying for the necessary documentation (a point stressed in sympathetic media reports of rogue construction). In Belgrade in 1987 according to building rules, a private individual would have to collect some ‘38 permits, certificates and declarations’ before construction began and in some cases additional documents were required. In the best-case scenario, applications were processed in six months but usually took much longer (Anojčić and Kačarević 1987). Thus, the linear life trajectory that socialism connoted (Crowley and Reid 2002, 7; Jansen 2015, 44) was compromised by housing shortages and opaque regulations. The main cause of illegal building was frequently cited in media reports to be the absence of spatial plans in most municipalities and that the authorities were failing to work sufficiently fast or effectively when spatial plans existed (Jambrović 1980).

If a citizen has to wait a year to determine the building conditions and then another six or more months for a building permit it is perfectly understandable that they lose patience and begin building as money saved will melt away [from inflation]. (Jambrović 1980)

Conversely, media also reported that the shortage of housing and eye wateringly high rental prices induced rogue builders to construct homes with the primary goal of renting out the space for a tidy profit on the grey rental market (Lydall 1989, 29). Economic crisis in Yugoslavia after 1979 likely increased both home based, informal production and broader entrepreneurial activity (as Pine [1996, 455] observes in Poland during the 1980s). In 1980s Yugoslav media discourses, however, speculation in the private property market was less prevalent than the omnipresent theme of the ‘usurpation’ of socially owned apartments (Archer 2015). The most dominant representation of rogue building was that of a task undertaken by workers out of necessity, frequently juxtaposed against the heartlessness of bureaucrats.^6^

Local authorities increasingly tolerated informally constructed homes provided that they adhered to the general urban plan. Though rarely explicated by the authorities, independent construction (in its legal, grey and illegal variants) actually cohered with a growing view that in the context of austerity measures in Yugoslavia during the 1980s (known as ‘economic stabilisation’) there was a need for citizens to contribute more of their own income [*lični dinar*] towards resolving their housing situation. By the early 1980s, there was also a broader acceptance of the necessity to provide the existing informal settlements with communal services like running water, sanitation and electricity (although the question of who would carry the financial burden – builders or local authorities – remained contentious). As a 1980 headline of tabloid *Večernje novosti*, observed there was now ‘also space for wild [construction]’ (M.L. 1980).

## The moral economy of access to housing

Lebowitz (2012, 137), drawing on the concept of moral economy as developed by Thompson (1971) and Scott (1976), argues that the concept of moral economy in real socialism was ‘not simply the inheritance of a traditional peasant society’ but rather emerged in accordance with a new social contract which saw the working class endowed with job security and improved living conditions in return for the acquiescence of their power in many other spheres (Lebowitz 2012, 132).^7^ The moral economy of the working class in real socialism could break with that of the vanguard (the party) when elements of this broad social contract were held to be deficient. Lebowitz extends the notion of the moral economy of the working class to workplace pilfering. In cases where one’s workplace put the worker in contact with scarce material resources it was considered acceptable behaviour for individuals to make use of these resources for networks of family and friends (Lebowitz 2012, 133; cf. Wedel 1986; Sík 1988b; Ledeneva 1998).

Keeping in mind Palomera and Vetta’s (2016, 3) recent observations that the moral economic approach can both advocate ‘a grounded understanding of the more abstract and global political-economy processes’ while historicizing ‘the everyday realm of observation by accounting for class-informed dispositions in a particular time and space’, I suggest that independent home construction can also be understood within a moral economic framework which highlights ‘the ambiguous logics and values that guide and sustain livelihood practices’ (Palomera and Vetta’s 2016). In discussing the various tactics and strategies of accessing suitable housing with oral history narrators, the custom of providing a home for oneself and family evidently supersedes the adherence to regulations like the legal duty to acquire advance planning permission for building a home. It also outweighs the potential risk of engaging in forbidden tactics to access construction materials via the workplace or taking extended sick-leave [*bolovanje*] from the workplace to undertake home construction projects. In cases whereby individuals attempted to engage in illicit activity in order to achieve these goals, this was broadly seen, both by narrators and dominant media discourses of the time, as legitimate (if not strictly legal). On the relatively rare occasions when rogue constructions were destroyed (entirely in accordance with the letter of the law and planning regulations) such destruction was widely seen as immoral, unfair – in the words of one protagonist, downright evil [*dušmanski*].

A key legitimizing factor in informal home construction is the harnessing of physical effort which resonated with dominant socialist discourse valorizing physical labour. Stressing one’s arduous, embodied labour (and that of friends, neighbours and family) served as an alibi for potentially illicit activities. If construction was undertaken in a subsistence-like manner, then it was necessarily considered legitimate (despite the sometimes ambivalent attitudes towards the construction of independent family homes on the part of the authorities). In this way, accusations about attempts to exploit profit in real-estate speculation could be deflected by builders through reference to the familiar and legitimate trope of physical labour, and the limited, subsistence nature of the construction (‘just for my family’, ‘to provide my two sons with a home’). As Jašarević (2007, 275) observes in her study of the differentiation between subsistence and the illegitimate and immoral accumulation of profit in the ‘Arizona’ market in Bosnia and Herzegovina, the social context not only referenced ‘the egalitarianism of the industrial, socialist past’ but also ‘reinvents the normative subsistence of the agricultural peasant tradition’. In discussing home construction, the same dualities were invoked. Narrators linked the pre-socialist tradition (imagined or experienced), of the patriarchal, rural homestead as a normative model for home construction. Yet according to their detailed descriptions of the construction process they also understand it as cohering with Yugoslav visions of socialist modernity and as such symbolically bridging the officially propagated dichotomy of pre-modern rural practices and the behaviour of modern, socialist, urban subjects.

Regardless of their infringement of laws (or, perhaps exactly because of this), many individual home builders consider their constructions in moral terms, emphasizing that their own savings and physical labour (as well as that of extended kin networks) went into the building process. This is contrasted with those less-deserving individuals who received a socially owned flat (funded by society as a whole). Some builders positively describe independent home construction in the normative language of Yugoslav socialism. Rather than considering themselves the victims of a bureaucratic ‘new class’ (Djilas 1957) who had taken the lion’s share of subsidized housing as urban sociologists like Živković (1981) and Vujović (1986, 1987) have inferred, builders recall generous credit from the workplace and favourable conditions for its repayment. Many workers remember the process in very positive terms and consider themselves very fortunate (particularly in comparison to the difficult position their children’s generation now find themselves in the mid-2010s). They describe the state as benevolent for assuming a passive role in facilitating such construction. As one builder stated, ‘While you saved for a house a lifetime would pass! You got credit, worked on it [the house], thank you state!’^8^

For those with higher earnings framing independent home construction as a moral endeavour was of even greater importance in order to deflect possible accusations of seeking excessive profit or engaging in illicit business like property speculation. By the 1980s the lively entertainment and tabloid media of Yugoslavia flagged the growing gap between ordinary Yugoslav workers – a demographic disproportionately affected by the austerity measures of the decade – and privileged politicians, footballers and celebrities.^9^ In this context Serbian pop-folk music performer Tomislav Čolović known as ‘Mali Mrav’ was featured in a spread in *Sabor* tabloid pop-folk music magazine about his two newly built houses in Kraljevo (‘one for each son’) and a holiday home on the Adriatic coast. He made reference to his difficult childhood and working-class origins, legitimizing his home constructions through his own physical labour and honesty. ‘I laid half the bricks myself. And I’m not ashamed. I worked honourably and honestly – I didn’t steal. Now that I have it, I want to enjoy it!’ (Pantelić 1988, 38–39).

## Destroying rogue constructions: the case of Staniša Simić

Despite a greater acceptance of the independent construction of family homes and the acquiescence of the authorities and workplaces in the 1970s and 1980s, ‘rogue housing’ was still sporadically destroyed by municipal and city authorities. Although most builders tended to adhere to para-legal urban plans to evade the wrath of municipal inspectors, certain structures were levelled because the land in question was to be used for another purpose (the construction of apartment blocks or public facilities) or in order to serve as an example to other builders.^10^ In 1978 media reports claimed that over 500 new constructions (houses, garages and sheds) were destroyed by officials who made a total of 2300 interventions in Belgrade, 10 times a day on average (Jović 1979). The most work was undertaken in the working-class municipality of Rakovica. In Kneževac, Resnik and Kijevo, peripheral neighbourhoods of Rakovica on Belgrade’s southern fringes, every sixth house was allegedly built ‘wildly’ (Jović 1979). But destroying rogue construction had little more than a symbolic effect. By 1987 conservative estimates held that over 30,000 unlicensed constructions existed in Belgrade, a threefold increase since 1975 (Anojčić and Kačarević 1987).

In late 1983, weekly tabloid *Novosti 8* investigated the case of Staniša Simić, a cook employed at the construction firm ‘Neimar’, who built an unlicensed house on the outskirts of Belgrade which was subsequently demolished by the municipal authorities of Palilula. Simić freely admitted to undertaking rogue construction in order to solve his housing situation. The sale of lots for licensed home construction in Belgrade was not occurring and the rules for construction were extremely opaque (Kordić 1983). Indeed reports at the time emphasized that for some builders it was more efficient and cost-effective to pay fines rather than to collect the necessary technical documents and pay the communal charges (*Novosti 8*, December 25, 1979). Simić and his wife spent four and a half years working in Iraq for a Yugoslav firm and saved 40,000 USD for a house. In Višnjica, a village above the Danube bank which by the 1980s was appended to Belgrade, he found a site. However, he was unable to obtain a building permit at the local municipality, Palilula. He waited for six months in vain. As *Novosti 8* reported: ‘From day to day the price [of materials] was rising, around his site houses appearing without building permits so Simić decided to join them. The homeless person [*beskućnik*]^11^ always hurries, even when the rules are not on their side’ (Kordić 1983). A 1980 headline in Croatian daily *Vjesnik* aptly proclaimed, ‘Life does not wait for spatial plans’ (Jambrović 1980).

Simić began building with the hope that he could arrange a permit retroactively. He adhered to the urban plans of the neighbourhood (anticipating that this would help with obtaining legalization) even employing a surveyor to assist with the planning of the house. ‘I didn’t want that tomorrow someone gives me grief and catches me out for some minor detail, so I strictly adhered to all the details that the urban planners foresaw for the area’ remarked Simić. ‘With the same hope, some 50 others built [unlicensed homes in the same area]’ (Kordić 1983). When his house was half complete, Simić received a fine from the local court to the sum of 500,000 Dinars but he claims the court did not expressly forbid him to build nor did it warn him of an impending demolition order. ‘I even told the judge that I would continue building!’ He paid the fine and continued finishing the house. Some of his neighbours received similar fines but also did not halt construction.

In June 1983, authorities from Palilula municipality announced that Simić’s house would be levelled but the bulldozers failed to arrive on the scheduled date. Simić suspected that the municipality was calling his bluff in order to levy more fines. A month later he received another demolition order. He complained formally but his objection was rejected and the levelling of the house was scheduled for October. But still the bulldozers did not come. Simić hurriedly moved in with his family. When he received the fourth demolition order on 31 October 1983, Simić realized that it was no longer an empty threat. Police, a building inspector, a bulldozer and a wrecking ball arrived to his home. He pleaded with the authorities to grant him one more day to salvage the roof tiles and load-bearing beams (valuable materials that could be reused) but they did not allow him. The house was immediately demolished (Kordić 1983). Simić took issue with the way in which the house was levelled.

If they had to destroy it, did they have to do it so evilly? Nobody has their house levelled like that; they always usually let the person take out the materials, not to totally destroy him. Nobody builds a house out of anger! (Kordić 1983)

The other houses in the neighbourhood, also loosely adhering to the urban plan of the area but without the necessary documents from the municipality, remained completely intact. All builders received demolition orders from the municipality but only Simić’s house was levelled.

The demolition of the house caused consternation in the neighbourhood amongst those who feared their own houses might be targeted next. Ljubiša Najdić, a farmer who owned land in the area and was also building illegally, mentioned that he was afraid, like other people (Kordić 1983). Najdić wished to build a house for his son and daughter – ‘what is wrong with that?’ His neighbour Vojkan had three houses, one for each of his three daughters. Najdić mentions that ‘everyone building is doing so out of necessity, nobody is building in order to sell’. The old settlers of Višnjica who were obliged to sell their land to the municipality under compulsory purchase orders claimed that the municipality was the one speculating, allegedly buying up land and reselling it for an inflated price (Kordić 1983). Stressing the immorality of state actors (at the municipal level at least) Najdić’s mother implored ‘how can they [municipal leaders] have the right to *both* flats *and* houses, while society neither gives us flats nor allows us to build independently?’ (Kordić 1983, my emphasis).

At the municipality of Palilula, journalists discovered that the house of Staniša Simić was demolished to serve as a warning to other builders. Allegedly other houses would be destroyed because the land was reserved for different purposes (although the purpose was not revealed; blocks of flats or public services like hospitals or schools were not planned). ‘They [rogue builders] cannot act like outlaws [*hajdučki*]’ representatives from the municipality claimed (Kordić 1983). However, just a week later reports emerged that officials of Palilula municipality had engaged in illicit housing activities of their own. Twelve small flats earmarked for social use were exchanged for four larger flats in the desirable Belgrade neighbourhood of Dorćol. These four flats were promptly gifted to four municipal functionaries from Palilula (N. O. 1983; Pavlović 1983).

Municipal staff were also implicated in the unlicensed construction of a restaurant and night club called ‘Amsterdam’ in Belgrade’s northern suburb of Borča (with some officials even attending the venue’s opening party). *Novosti 8* reported on the hypocrisy of the Palilula municipal officials who acted ‘cruelly’ to those without connections like rogue builder Staniša Simić who are ‘far from their heart and armchair’ while facilitating the illegal activities of those with connections (N. O. 1983). Staniša Simić worked for a Yugoslav firm in Iraq, a legitimate destination which in the early 1980s was a hotspot for Yugoslav construction, in order to save for his home. The proprietor of ‘Amsterdam’, however, was a returned guest worker [*gastarbajter*] who had profited in the capitalist West and was now investing in a private hospitality business – a restaurant and nightclub with immoral and hedonistic connotations.

## Building socialism? A (nearly) legal housing biography

The case of Staniša Simić is somewhat remarkable in that his house was actually destroyed. Of some 30 narrators informing this article, most of whom had participated in independent home construction, no individual recalled the demolition of a ‘rogue property’ within their circle of family, friends and neighbours. The following section follows the housing biography of a family with roots in Croatia who now live in the Belgrade suburb of Resnik (in Rakovica municipality). Unlike the case of Staniša Simić, the construction of the family home in Resnik was carried out in accordance with (most) rules and regulations. Two elderly sisters, Dragica and Nada, form the nexus of this extended family network along with Dragica’s husband Pero and the deceased Mladen, Nada’s husband who passed away in 2012. While Nada and Mladen had lived in Belgrade since early adulthood, Dragica and Pero lived in Čepin, a village-cum-suburb of Osijek in Slavonia (Eastern Croatia). After the murder of members of their extended family and neighbours in Slavonia following the outbreak of war in 1991, Dragica and Pero fled to Serbia and later lived in Serb-occupied Vukovar.^12^ Their housing biography was thus caught up in the 1991–1995 War in Croatia and reintegration of Eastern Slavonia to Croatian sovereignty in 1998. Since 2004 they live permanently in Resnik, in a house whose construction began in the early 1970s, the brainchild of the two sisters and their long-deceased mother.

Dragica and Pero met in teacher training college in Zagreb in the late 1950s. Both were born a few years prior to the outbreak of World War II. As a child, Pero and his family were refugees in Western Serbia, returning to their home village in Slavonia with the end of hostilities. Dragica and her sister Nada were born in the village of Čovac, near Okučani in Western Slavonia. Their father had been killed during the war and their mother was one of some 80 young Serbian widows in the small village, separated by a swampy forest from the former Ustaše concentration camp of Jasenovac. The young couple moved to Čepin upon completing their teacher training and took up work in the village school. They received a small flat to use, a rare privilege for young teachers to be immediately granted. As luck would have it a colleague left the school just before they arrived, his flat became vacant and no other staff member wanted to take it. Dragica and Pero moved in to the flat with just ‘two suitcases’ using credit to furnish it and install a functional kitchen.

Pero had three brothers living in Eastern Slavonia. Dragica describes how ‘all of them built, they started to build houses, so we did the same. If they are doing it then why not us too! … we had the least money but lots of will and strength’.^13^ And so, Pero and Dragica began construction on the house in Čepin through the use of favourable credit obtained via their workplace. The decision to build a family home was not dictated by necessity alone but of the spirit of the times; that ‘everyone else’ was doing it, ‘there was this atmosphere’.^14^ Additionally, Dragica mentions the ‘patriarchal attachment to the family home’. Her sister Nada interjects, ‘It was simply the mentality, we all grew up in houses, were born in them, there was a need that a person has *their own* house’^15^. As Pine (1996, 456) stressed with regard to homes in Górale, Southern Poland, official discourse favouring apartments coexisted with local practices centred on houses. She notes that in a centralized state like socialist Poland the house had the capacity to adapt and endure (Pine 1996). In a more decentralized context like Yugoslavia such a capacity was arguably far greater. Traditional models of home building could be adapted within the socialist context and negotiated with workplace and municipal authorities.

Pero recalls how workers in Osijek took credits to build houses ‘but always according to a system where one would help the other […] This would never be completed in a year, it would take many years to build a house to completion.’^16^ While these semi-rural family home-making processes may reflect patriarchal kinship patterns (Halpern 1986; Halpern, Kaser, and Wagner 1996; Kaser 1996, 2008) and were dependent on the reciprocal exchange of cooperative labour (Sík 1988a, 1988b; Brunnbauer 2000) they were conditioned by Yugoslav socialist modernity and articulated in its vocabulary. The skills that workers acquired through new vocational education opportunities and factory training were put to use as was cheap credit from the self-managing workplace.

Rather than considering independent construction as a deviation from the socialist ideal mode of living, Pero, then an active party member, stressed the collective nature of the building endeavours in which he was a participant in the outskirts of Osijek and Belgrade. Not only had builders constructed their homes but also (allegedly) much of the surrounding infrastructure. Pero explained how the extended family and their neighbours in Resnik, Belgrade, built up ‘the entire infrastructure of this street ourselves, it was not financed by anyone else. We bought slabs [for the street], we asphalted, we installed water, sewerage system … the only thing which we didn’t pay for or build was street lighting.’^17^ The relatively flexible tenets of Yugoslav self-management thus enabled builders to make claims that they were legitimately ‘building socialism’ in ways which would have been less credible in Warsaw Pact countries.

Hierarchies existed in independent construction as evident from the recollections of Dragica and Pero. Neighbourhoods built up by independent builders were imbued in varied degrees of cultural and economic capital. While *gastarbajteri*, returning migrants from Western Europe (Bernard 2012; Ivanović 2012; Le Normand 2016), may have had economic capital to invest in home construction in the eyes of the teachers they were lacking not only in cultural capital, but also allegedly in hygienic practices according to Dragica.^18^ The division of their expanding village-cum-suburb into more and less desirably parts was evident to the teachers. Dragica and Pero recall one settlement in Čepin which had the nickname ‘Nemanovca’ (*Moneyless*) – ‘those without money build houses there’.^19^

Dragica describes how one day she and her husband Pero were walking in Čepin and happened upon a former pupil in Nemanovca. He had been a problematic boy but had succeeded in finishing elementary school and continuing to complete basic secondary schooling. Dragica asked how he was doing and he responded, ‘Great, I am building a house here – enter and have a look!’ Upon entering Dragica encountered a gaggle of her former pupils. They proudly told the teachers that they were all now married and had received cash in lieu of wedding presents. With that money, they could buy the sites and dig the foundations, perhaps even afford the basic frame for the house. The former pupils, mostly tradesmen, then explained the division and exchange of their labour: ‘one colleague is a plumber, he will do the pipes. Another colleague is an electrician, he will do the wiring, and so five or six tradesmen build the house’.^20^

In 1971 Dragica’s ailing mother came to live with Dragica and Pero in Osijek having sold her home in Western Slavonia. In consultation with her two daughters she decided to use the proceeds to build a house. Dragica’s sister Nada lived in Belgrade with her husband Mladen. The two were subtenants paying expensive rent for rather insecure accommodation. Private rental or subletting [*podstanarstvo*] was nearly entirely unregulated in Yugoslavia creating a situation of insecurity and exploitation for the many that rented (Petrović 2005, 175). The lack of privacy (with landlords often living in the same building or house), high cost and insecurity (regular demands of advance payment of up to a year with no legal recourse should eviction occur) and poor material conditions (the lack of an indoor bathroom or running water) were cited as perennial problems by narrators. While renting, efforts were usually made to access suitable long-term housing through other means.

Mladen was employed in the Veterinary faculty of Belgrade University as an assistant but had little chance of receiving a socially owned flat for at least a decade. He and Nada found private-rented accommodation in a suburb of southern Belgrade (Braće Jerković) in a basement flat where they stayed for two or three years. Nada deemed it ‘quite expensive’ in relation to their pay claiming that ‘when one calculated how much we were spending annually on rent it was a better solution to build a house, to take credit and pay it off … So it began’.^21^ The sisters, with their mother’s blessing, used the sale of the family house in Western Slavonia to buy a site in Resnik, on the southern fringes of Belgrade. The plan to build a family home was enthusiastically accepted by their husbands. Deda Nikola, Nada’s father-in-law, was a skilled builder and he led the construction project.

## Connections and privatized physical labour in home construction

Although their home in Čepin, Osijek was sufficient, Dragica and Pero were also involved in the project of constructing the extended family home in Resnik where they maintained a ‘proxy’ presence (Dalakoglou 2010) after the 1972 purchase of the site. The building of the family home demanded a huge amount of physical labour and time from all of the family members involved in its construction. Unlike Verdery’s (1996, 39–50) conceptualization of the ‘etatisation of time’ whereby the socialist state would impinge upon and capture the time of its citizens, in Yugoslavia such embodied labour was increasingly *privatized* from the 1960s onwards. House building saw individuals like Nada and Dragica and their extended family networks investing a great deal of their free time and physical effort in construction in such projects.

In Belgrade, Deda Nikola and Mladen kept typical socialist working hours in their paid jobs (working from approximately 07:00 to 14:00) and would return to work on the house in the afternoon. Deda Nikola worked as a foreman in a building site in the nearby Rakovica suburb of Vidikovac and so ‘he understood all types of building’. Nada also worked hard on the house and Pero and Dragica would travel from Osijek to participate in the construction during the weekend ‘which of course for them was a great burden’ Nada recalls. ‘Instead of relaxing (and in addition they had a house that they had built there, not yet fully completed) they would work’. They would drive from Osijek on Fridays, ‘not every weekend, but often’.^22^

Pero describes how he familiarized himself with the city of Belgrade by driving around it with his brother-in-law Mladen in order to arrange the purchase of construction materials. Despite the relatively favourable conditions of the 1970s for builders (which included ample workplace credit, porous municipal regulations and free time in the afternoon and weekends to engage in building largely unhindered by the party-state), the context that building occurred in was nonetheless a (market-)socialist economy of shortage and access to building materials was unpredictable. Pero describes problems in finding materials due to the sheer amount of independent building taking place at that time. ‘There were shortages then … Particularly cement. You needed connections (*veze*), to bribe, to get cement’. Nada added, ‘but we always had good connections! […] the connection was the husband of my cousin in Split who worked in “Dalmacijacement”’. Of course Deda Nikola, who worked as a foreman in a Belgrade construction firm, could also be counted upon to source materials from his workplace and could request that his workers form and shape particular items (for example, iron frames). All narrators negated any accusations of financial impropriety pointing out that ‘all materials were paid for! … Nothing was dishonourably gained!’ (although they conceded that the labour of workers who processed the materials, Deda Nikola’s staff, was not remunerated).^23^

Displacing the ambiguity of accessing ‘connections’ and (potentially) engaging in illegal behaviour, an emphasis was placed on the arduous physical labour that individuals undertook. According to a moral economy of housing construction, certain infringements on rules and regulations (at the workplace, of the municipality, planning regulations) were deemed legitimate in the quest for working families – normative socialist subjects – to access housing. As Ledeneva (1998, 168) writes in the context of Russia, ‘to obtain something by *blat* [connections] – in modest volume, with discretion, normally in situations of urgent need and within a closed personal circle – is a norm; to exceed limits is theft, corruption … ’. In Yugoslavia, a similar understanding existed. In fact, it would probably have been considered highly immoral of Deda Nikola *not* to have used his workplace to source items in shortage for his family members. Similarly, if Nada’s cousin in Split did not help the family access cement this would also have been viewed by the family as immoral; a failure to reciprocate.

Nada, Dragica and Pero went to pains to stress that their home construction, both in Osijek and Belgrade, was entirely legal and adhered to all applicable rules and regulations. Nearly … Nada explained that the kitchen we were sitting in was actually temporary construction which was supposed to be razed once the house was completed in the 1970s. She states, however, that the family was not in a material position to finish the house and thus required continued use of the space. She and her late husband received a demolition order from the municipality of Rakovica in the mid-1970s. Rather than knocking down the kitchen as demanded, Nada and Mladen appealed to the municipality and consulted with a legal clerk who told them: ‘It’s like this, I just began in this position, for sure I will be at least a couple of years here. If you sign that you will knock the temporary structure down when you complete the house, while I am here the document will remain in a drawer, you will let me know [when the temporary kitchen is demolished].’^24^ The municipality never followed up the case and the temporary structure was never razed. It remains as the kitchen, the centre of social life for the three generations who now live in the house.

In home-building projects, various individuals (including distant relatives, friends, colleagues and neighbours) were prepared to part with their time and physical labour, in a reciprocal manner. The hiring of professional labour was kept to an absolute minimum with some narrators referring to ‘stealing the craft’ of electricians and plumbers as they worked on a short-term basis^25^ (that is, learning from their work through observation. cf. Creed 1998, 201). Nada stresses that ‘the main work, physical works, we did ourselves’. Pero adds with a good humoured laugh that ‘even the children had to work, they had to fix 50 crooked nails before going out to play!’ Marija, Nada’s sister-in-law, recalled similar arrangements of the harnessing of reciprocal labour in independent home construction. She and her late husband built a house in Čačak during the 1980s with his family. She recalls the experience of neighbours, family and friends and how they invested collective physical efforts in its construction:

Everyone was together […] when the foundations were laid or the main panels placed there would be 20 people working, just one skilled labourer but 20 volunteers … and of course it was necessary to cook for all those people. […] the youngest men worked on the concrete, the most difficult job, lifting sand bags. There were no cement mixers then, two men mixed it and poured it into the foundations […]^26^

In suburban tracts of contemporary Serbian cities in the 2010s, the reciprocal exchange of cooperative labour is no longer salient as it had been in late socialism. Marija laments the demise of the moral economy of housing construction, ruing the changes that have taken place in the Serbian labour regime since the end of socialism.

It is unimaginable that that would happen now in Serbia. Then, people worked from 6 h to 14 h but now it would not be possible with the [contemporary] working hours. Those who work could not do it, those who do not work would refuse to work for free. The situation has changed […] People used to take their annual holidays for building. For the most demanding [*udarni*] jobs like laying the foundations or the floors that have to be done in one day, from dawn to dusk, a large number of people need to be present.^27^

Physical labour was associated with hard work even when it was undertaken to the detriment of the workplace (for example being absent from work to build a house or pilfering materials to be used in home construction). The concept of ‘hard work’ envisaged by narrators was sufficiently elastic to encompass a broad range of tasks unrelated to one’s waged labour but undertaken for the common good of the family and community. This included independent house construction but also the production of agricultural products or participation in the harvest in one’s place of origin.^28^

## Accessing credit (to Iraq and back)

Workplace credit was the most common way of financing independent home construction (sometimes also helped by the sale of an inherited property like that of Nada and Dragica’s mother which financed the purchase of their site in Belgrade). While credit served to somewhat mitigate the exclusion of many workers from the system of socially owned housing provision, it favoured better paid workers and thus forged social inequalities of its own (for example, the hierarchy of working-class settlements like ‘Nemanovca’ (‘moneyless’) outside Osijek compared to the slightly better-heeled community of self-built houses where Dragica and Pero lived). When it came to the distribution of credit for housing through the workplace, candidates who could offer the most financial participation – richer workers – were favoured. Vušković (1976, 37) writes of the distribution of credit for housing purposes in Split during the 1970s noting that highly skilled workers and those with high professional qualifications received ‘seven times more credit than un-skilled and semi-skilled workers, four times more than those with low professional qualifications and three times more than those with middle professional qualifications’.

Marija, Nada’s sister-in-law and former employee of Kvarc construction materials firm in Mladenovac, described the construction of the family home in Čačak. Unlike most of the other narrators, Marija and her husband enjoyed the privilege of a socially owned flat. Already in possession of a home, the couple could not obtain the most favourable loan for housing with 3% rate of interest but instead took a commercial credit of 10,000 Deutsch Marks with 7% interest over 20 years to build a 200 square metre house for Marija’s mother-in-law and extended family in Čačak in 1982.

In the beginning it was difficult; we gave my entire pay for the repayments. We skipped one repayment to go on summer holidays (I worked in accounting so I could swing it). After four or five years, inflation started to eat away at the repayments. We paid it off in seven or eight years.^29^

The house was completed by 1986.

Marija’s elder sister Slobodanka, and her husband Dragan, similarly made use of credit for home construction but their experience was much more arduous. Slobodanka may have had the opportunity to receive a socially owned flat through her employer, pharmaceutical manufacturer ‘Galenika’. This firm was a more successful one than her husband’s and thus more capable of providing flats for its workers. Dragan could expect to wait some 15–17 years to access a socially owned flat from his firm. Already nearly 40 years old, this was a lengthy prospect. Another possibility was for the firms of Slobodanka and Dragan to jointly contribute to a socially owned flat – this might have been achieved in less than 10 years. Dragan was adamant, however, that he would build a house independently. Like the unfortunate protagonist Staniša Simić whose house was destroyed, Dragan travelled to Iraq with his firm in search of higher wages. He spent four years working in Iraq between 1976 and 1986 (receiving additional compensation when the Iran–Iraq war broke out). The construction of their family home in Belgrade’s southern fringes began in 1979 and it was sufficiently completed for the family to move in on 1 May 1982.

In addition to Dragan’s stints working in Iraq, the couple made use of multiple credits to construct their home. Upon buying the site and obtaining a building permit, Dragan could access credits from the bank and his firm:

In those days the credits were favourable, I think we got 42 million dinars from the bank and it all was spent on the house. Then my wife accessed credit from her firm and got 5 million (with that we bought the windows) and I got 10 million from my firm and the rest I had to finance. But I still have not finished the house! The top floor still remains to be completed. For my son or daughter, if they want to build … ^30^

He describes how interest was very favourable for individual construction. Interest rates for housing loans ranged from 3% to 4% for bank loans and 2% for loans from the social sector enterprise. Dragan received a 20-year bank loan and a 10-year loan from his workplace. He details the nature of repayments and the impact of inflation had on the finances of individuals. Like Marija, he recalled that it was difficult to meet the repayments in the first year or so. However, each year inflation would be at least 4% (usually far higher) reducing the costs of repayments on an annual basis.

After ten years, the credit was so worthless that the debt collectors called to ask me to pay it off at once because the value of the credit fell to the cost of a packet of matches. It was more expensive for them to perform the administration.^31^

Credit for home construction was not revalorized during most of the 1980s. Dragan believes that everybody planned taking credit (and thus their home construction) around inflation. ‘That was the politics, I don’t think the state organs were so stupid … that was the system, that the masses, ordinary people [*raja*, *obični narod*] can get to a home.’ But not all ordinary people could access sufficient credit to construct a home. Solidifying the divide between those who could and could not access credit became more evident when the repayment of credit was made even more advantageous due to the rampant inflation of the 1980s (Magid 1991, 42–43). Those individuals who took credit during the early 1980s (like Marija) saw the real costs of unadjusted repayments shrink massively. With the sustained economic crisis, however, credit was being squeezed. Before 1984 cheap consumer credit served many Yugoslav households. The interest rate was usually around 12% but with inflation approaching 55% the price of repayment dramatically reduced (*Ekonomska politika*, 23 April 1984). IMF-imposed stipulations required interest rates to approach real levels of inflation. This was achieved by April 1985 rendering credit a more difficult means of income supplementation (Magid 1991). By 1987 credit for housing began to be revalorized and in the last years of Yugoslav socialism the scope for using it to its full inflationary advantage was greatly reduced, though not fully extinguished (Anojčić and Kačarević 1987).

## Conclusion

This article has sought to demonstrate the moral and temporal logic of independent home construction by Yugoslav workers in late socialism, exploring some of the ways workers valued their actions and constructed claims to make their cases legitimate and understandable in a wider social context. Although workers pursued various means to access suitable housing in an economy of shortage such strategies were underpinned by ‘a popular consensus as to what were legitimate and illegitimate practices’ (Thompson 1971, 79). The moral economy of home building between the state and the citizen was not coherently set in explicit rules and encompassed implicit allowances for flexibility. Provided that home building was undertaken to house one’s family and not (at least in the initial stages) aimed at generating profit thorough subletting, it could be viewed as an inherently positive activity by communities of informal builders and tolerated by the authorities. The embodied labour that home construction implied resonated with discourses of self-managing socialism. Stressing the investment of physical effort in the construction process deflected counter-claims that independent house building was illegitimate or illegal.

The patriarchal notion of a family homestead continued to hold weight for workers and the conditions of late Yugoslav socialism were amenable for family home building by means of the reciprocal exchange of cooperative labour, albeit in a modernized form which was heavily influenced by the self-managing workplace and new social mores. For example, sisters Nada and Dragica and their families constructed a family home in Resnik with explicit reference to the pre-socialist memory of a patriarchal village home. Despite its patriarchal underpinnings, however, this was a female-led building project – Nada and Dragica’s mother financed the site and were instrumental in the planning and construction of the home. Similarly, the neighbours of Staniša Simić were building houses for their daughters as well as their sons. Although female emancipation was a ubiquitous tenet of state-socialism which scholars like Penn and Massino (2009, 2–3) observe was ‘often more strategic than genuine’, tending to reinforce rather than challenge essentialist notions of gender, in the case of informal housing practices a modicum of progressiveness was evident.

In addition to the widespread belief that working families were entitled to bend or break rules to access a home, particular temporal configurations can also help us better understand aspects of independent construction and how such activities were undertaken by protagonists. Rising prices of property and materials coupled with high inflation, particularly after 1978, served as an impetus to act as quickly as possible when building a home. The authorities, particularly at the municipal level, served to slow this process down through unclear and inconsistent bureaucratic practice. A rogue construction needed to be undertaken even faster than usual and was particularly reliant on the reciprocal exchange of cooperative labour (cf. Sík 1988a, 602). The more advanced a house was, the lesser the chance was that it would be razed and so it was imperative to undertake the initial work at great speed. In order to evade the authorities, construction might commence late in the evening and last through the night or during the weekend and holidays when the authorities were less likely to intervene. Builders also were aware that in practice, once a house was built and remained standing for a number of years, the prospect that it would be demolished would diminish. Construction thus took place in linear spurts of movement, influenced by cyclical processes (such as occasions where municipalities temporarily cracked down on rogue builders as well as times when authorities were more permissive during the cold winter months).

A contemporary resident of the settlement of Kaluđerica, Belgrade’s best known self-built community,^32^ believes that many of the builders of homes during the 1970s and 1980s ‘grasped the right time to act and were proven right – their children could never do so now’.^33^ They acted pragmatically, not without risk, according to a rather fleeting permutation of circumstances which included the decentralization and weakening of state authorities, inflation which was reducing credit over time, access to physical labour through family networks, and conditions which began to favour such entrepreneurial practice provided that it was framed in the appropriate language of Yugoslav socialism and undertaken according to the prevailing logic of the moral economy of home building.

Networks of neighbours, families and friends constructed and transformed the suburban fringes of Yugoslav cities like Resnik and Kaluđerica in Belgrade and Čepin outside Osijek. Such working-class communities, largely forged through the embodied labour of their inhabitants, emerged with the intersecting of the enduring reciprocal exchange of cooperative labour with the conditions and institutions of self-managing socialism. The home-building activities of these communities combined with situational and contingent forces like workplace credit, more permissive rules and a deferral to the market in the 1980s started to revolutionise broader property ownership concepts in Yugoslavia, legitimizing self-built homes and bringing this model of home construction from the marginal to the mainstream.

## Notes

1. Although Belgrade is the focus of the research many narrators had roots in other parts of Yugoslavia. All narrators were resident in Belgrade when the research was undertaken in 2014 but many life histories were informed by previously living and working in Yugoslav locales as diverse as Prizren (Kosovo), Nikšić (Montengro), Rijeka Osijek and Knin (Croatia), Čačak, Subotica and Lazarevac (Serbia) as well as stints abroad in Germany, Iraq and the USSR. All narrators are referred to by pseudonyms. For a more detailed overview of the methodology employed, see Archer (forthcoming).

2. Interviews with Dragan, Pero, Nada and Dragica. Belgrade, 2014.

3. Interview with Miroslav. Lazarevac, 2014. Interviews with Slobodanka, Ljiljana, Mirjana and Gordana. Belgrade 2014.

4. Interview with Branimir. Belgrade, 2014.

5. Particularly after the 1976 Law on Associated Labour, decision making (including the provision of credit or socially owned flats) was devolved to the basic units of associated labour, the smallest units of a socially owned company which were legal entities.

6. For example, see: N. O. 1983. ‘Amsterdam’ pod zaštitom.’ *Novosti 8*, December 17.

7. On the similar concept of ‘welfare dictatorship’ see Jarausch (1999).

8. Interview with Nada. Belgrade, 2014.

9. For example, see Hofman and Sitar (2016) and ‘O nama i odgovornosti. Dragoslav – Draža Marković odgovora radnicima’ 1984. *Beogradski radnik*, no. 3, January 25.

10. During the 1960s Belgrade municipal delegates identified cases whereby rogue builders would deliberately build a home on a site earmarked for a future development as a strategy to secure a new apartment as compensation (Le Normand 2014, 157).

11. Homeless in the Yugoslav socialist context did not usually mean living on the street without shelter but tended to refer to substandard and precarious living conditions including cohabiting with relatives in cramped conditions, squatting common areas of a socially owned apartment building (usually attics or basements) or renting on the grey real estate market from exploitative landlords, a phenomenon Tsenkova (2009, 29) terms ‘hidden homelessness’ in socialist states.

12. Pero was mobilised in 1993 to the area surrounding Vukovar (‘Thank God that by that stage the UN forces had arrived and there was not, how can I say, military action, and the situation was calming itself. All that dirty work of 1991 had already vanished … ’) and Dragica found work as a teacher. They remained in Vukovar for 14 years, returning to Resnik upon retirement. (Interview with Pero. Belgrade, 2014).

13. Interview with Dragica. Belgrade, 2014.

14. Interview with Dragica. Belgrade, 2014.

15. Interview with Nada. Belgrade, 2014.

16. Interview with Pero. Belgrade, 2014.

17. Interview with Pero. Belgrade, 2014.

18. Interview with Dragica. Belgrade, 2014.

19. Interview with Dragica. Belgrade, 2014.

20. Interview with Dragica. Belgrade, 2014.

21. Interview with Nada. Belgrade, 2014.

22. Interview with Nada and Pero. Belgrade, 2014.

23. Interview with Nada and Pero. Belgrade, 2014.

24. Interview with Nada. Belgrade, 2014.

25. Interview with Branimir and Zora, Belgrade, 2014.

26. Interview with Marija. Belgrade, 2014.

27. Interview with Marija. Belgrade, 2014.

28. Interviews with Mirjana and Gordana. Belgrade, 2014.

29. Interview with Marija. Belgrade, 2014.

30. Interview with Dragan. Belgrade, 2014.

31. Interview with Dragan. Belgrade, 2014.

32. For a study on the settlement of Kaluđerica see Saveljić (1989). A critical artistic and activist interpretation of the production of knowledge about Kaluđerica has been compiled by Džokić, Neelen, and Milikić (2012).

33. Interview with Nenad. Belgrade, 2012.
